# Prevalence and quality of temporomandibular disorders, chronic pain and psychological distress in patients with classical and hypermobile Ehlers-Danlos syndrome: an exploratory study

**DOI:** 10.1186/s13023-023-02877-1

**Published:** 2023-09-19

**Authors:** Leon Willich, Lauren Bohner, Jeanette Köppe, Jochen Jackowski, Marcel Hanisch, Ole Oelerich

**Affiliations:** 1 Department of Prosthodontics, University Hospital Münster, Albert-Schweitzer-Campus 1, Building W30, Münster, D-48149 Germany; 2 Department of Oral and Maxillofacial Surgery, University Hospital Münster, Münster, 48149 Germany; 3 Institute of Biostatistics and Clinical Research, University of Münster, Schmeddingstraße 56, Münster, D- 48149 Germany; 4 Department of Oral Surgery and Policlinical Ambulance, Faculty of Health, Witten/Herdecke University, Alfred-Herrhausen-Str. 45, Witten, 58448 Germany

**Keywords:** Ehlers-Danlos syndrome, Rare diseases, Temporomandibular disorder, Oral health, Psychological impairment

## Abstract

**Background:**

The Ehlers-Danlos syndromes are a group of clinically and genetically heterogeneous hereditary diseases affecting the connective tissue. They are characterized by hypermobility of the joints, hyperextensible skin and friable tissue. According to current classification, 13 subtypes can be distinguished, of which the hypermobile and the classical subtype are the most prevalent. This study aimed to evaluate patients with classical (cEDS) and hypermobile (hEDS) Ehlers-Danlos syndrome regarding temporomandibular disorder (TMD), chronic pain, and psychological distress.

**Methods:**

Support groups from Germany, Austria, and Switzerland were contacted, and social media were used to recruit participants. Free text questions, the German version of the Depression Anxiety and Stress Scale (DASS), and the German version of the Graded Chronic Pain Status (GCPS) were used.

**Results:**

259 participants were included (230 hEDS/29 cEDS). At least 49.2% of the participants had painful or restricted jaw movements, and at least 84.9% had pain in the masticatory muscles, with 46.3% already having a diagnosed TMD. Multivariate analysis showed a significant correlation between TMJ involvement and chronic pain with a 2.5-fold higher risk of chronic pain with a diagnosed TMD. 22.8% of participants had a critical score for depression, 53.3% had a critical score for anxiety, and 34.0% had a critical score for stress.

**Conclusion:**

There is a high prevalence of TMD problems and chronic pain in patients with cEDS and hEDS. The lack of knowledge about these problems can create psychological distress. More research is needed to provide adequate treatment for patients with EDS.

**Supplementary Information:**

The online version contains supplementary material available at 10.1186/s13023-023-02877-1.

## Background

Ehlers-Danlos syndromes (EDS) are classified as rare diseases. EDS are a group of clinically and genetically heterogeneous hereditary disorders affecting the connective tissue. Characteristics are hypermobility of the joints, over-stretchable skin, and friable tissue [1]. According to the current classification, 13 subtypes can be distinguished [1]. Diagnosis of each subtype is based on clinical criteria and, in most cases, molecular confirmation. For each subtype, primary and secondary diagnostic criteria are defined, which are supplemented by laboratory findings as far as possible [1, 2]. New genetic techniques such as next-generation sequencing (NGS) can support clinical diagnosis and identify the genetic basis for the different types of EDS. Meanwhile, genetic analysis is essential to confirm or modify the clinical diagnosis of EDS. Currently, hEDS can only be diagnosed by matching clinical symptoms and excluding other subtypes by genetic testing, as there is no genetic testing for hEDS itself [3].

Recent studies estimate the combined prevalence of hypermobility spectrum disorders (HSD) and hEDS to be 1:500, suggesting that hEDS may not be a rare condition at all. At present, it is not possible to precisely report distinct prevalence rates for HSD and hEDS [4].

Classical EDS is inherited in an autosomal dominant manner and is characterized by considerable locus heterogeneity. Clinically, it is characterized by marked extensibility and fragility of the skin and joint hypermobility. Hypermobile EDS is also inherited in an autosomal dominant manner, although the underlying genetic defect is unknown. It is characterized by moderate extensibility of the skin, lack of brittleness, and marked hypermobility of the joints [4, 5]. The current classification differentiates between hEDS, where all diagnostic criteria must be met, and HSD, where some but not all diagnostic criteria are met.

Affected individuals often suffer from pain, in many cases even from chronic musculoskeletal pain [6–8]. As the disease progresses, neuropathies and central sensitization of pain signals develop, causing about 90% of affected individuals to suffer from chronic pain [9]. Few studies on treatment modalities make it challenging to guide treatment management for patients with EDS and chronic pain [10, 11].

According to current literature, patients with EDS are inherently more likely to suffer from temporomandibular joint (TMJ) problems [12–16]. Furthermore, a positive relationship between temporomandibular disorder (TMD) and generalized joint hypermobility has been demonstrated [14, 17–19]. TMD is defined as a group of craniofacial pain disorders, affecting the masticatory musculature, the temporomandibular joints or related tissue structures [20]. The frequent prevalence of TMJ problems in patients suffering from EDS explains the disease’s effect on oral structures and collagen. However, the exact nature of this relationship remains unknown [21].

Recent studies have shown that oral health-related quality of life (OHRQoL) is worse in people affected by EDS [22]. However, this lower OHRQoL does not correlate with lower objective oral health [23]. In addition, long diagnostic pathways to detect EDS were a common problem for affected individuals.

This study aimed to evaluate the prevalence and quality of TMD in affected people with cEDS and hEDS. Since the effects of pain and pronounced TMJ problems often lead to a reduction in OHRQoL, this is another step in studying this disease and developing treatments.

## Materials and methods

Data were collected from 01.02.2022–15.05.2022 via an online questionnaire in the German language. The study was approved by the Ethics Committee Westphalia-Lippe and the University of Münster (2022-005-f-S).

The study reports recording to STROBE statement and checklist for cohort studies.

### Participants

Participation was open to individuals over 18 years of age affected by EDS. Participants had to confirm that they had been diagnosed with either the classic (cEDS) or hypermobile (hEDS) subtype. As the survey was anonymous, the diagnosis was confirmed solely by the patients’ self-reporting. Participation was voluntary, and no incentives were offered. An electronic consent form was obtained online before the survey began. Participants were subjected to an online survey which was made available to affected individuals via support group mailing lists in Germany (Eherls-Danlos Selbsthilfe e.V. – www.bundesverband-eds.de, Deutsche Ehlers-Danlos Initiative e.V. – www.ehlers-danlos-initiative.de), Austria (SHG Ehlers-Danlos-Syndrom – Sozialinfo Wien - https://www.wien.gv.at/sozialinfo/content/de/10/InstitutionDetail.do?it_1=2099377) and Switzerland (Themenliste | Selbsthilfe Schweiz - https://www.selbsthilfeschweiz.ch/shch/de/selbsthilfe-gesucht/themenliste~thema~Ehlers-Danlos-Syndrom~.html). In addition, social media was used to disseminate the study further. Only fully answered questionnaires were included in the data collection. The survey questions focused on demographics, age, and gender. In addition, the questionnaire covered the diagnosis and symptoms of EDS as well as their temporal relation, general health, and dental health. Furthermore, specific questions were asked about pain in the TMJ and TMD and their diagnosis and therapy. A diagnosed TMD was confirmed by patient self-report. However, a confirmed TMD diagnosis according to the 2017 classification was explicitly asked for. A translated version of the questionnaire can be found in Additional file 1.

### Assessing psychological stress factors

The German version of the validated Depression Anxiety Stress Scale (DASS) was used to assess psychological stress factors in patients with cEDS and hEDS [24].

The questionnaire contains 21 items, of which seven are used to query the categories of depressiveness, anxiety, and stress. There are four possible answers from 0 (“did not apply to me at all”) to 3 (“applied to me very much or most of the time”) to choose from. The sum of the answers is calculated, whereby each of the three categories was considered by itself. According to current data, the threshold value for increased likelihood of depression and stress is ten, and for anxiety, six.

### Recording of pain-related impairment

To measure the extent to which participants are affected by pain in their daily lives, the German version of the Graded Chronic Pain Status (GCPS) was used, “Graduierung chronischer Schmerzen” (GCS) [25].

The GCS consists of seven questions — four related to different areas of pain-related impairment and the remaining three pertaining to pain intensity. The questions are answered using an eleven-point estimation scale ranging from zero (“no impairment”) to ten (“I was unable to do anything”).

For the evaluation, the scores obtained were converted into impairment points from zero to three, whereby initially, only the questions on pain-related impairment were weighted. The assessment of the questions on pain intensity only took place if the sum of the impairment points was less than three. Finally, the impairment points were assigned grades from one to four. Clinically, grade one was classified as “low disability – low intensity”, grade two as “low disability – high intensity”, grade three as “high disability – moderately limiting” and grade four as “high disability – severely limiting”, respectively.

### Statistical methods

The study was conducted to be fully explorative. Therefore, no power analysis was done a priori and all results were interpreted as hypothesis generating.

The data collected from the completed questionnaire were analyzed descriptively. To evaluate differences between participants with and without a TMD diagnosis, categorical variables were analyzed using a Chi-square Test. Continuous variables were analyzed using a Mann-Whitney U Test. All tests were performed at a significance level of α = 5%. A multivariable analysis was performed to calculate the impact of gender, age, time between first symptoms and diagnosis, membership of a support group, diagnosis of TMD, and frequency of annual dental visits on chronic pain. Statistical analyses were performed using IBM SPSS Statistics for Mac, Version 28.0.1.0 (IBM Corp., Armonk, NY, USA), SAS software V9.4 (SAS Institute Inc., Cary, NC, USA), and RStudio Version 2022.07.1 + 554 (RStudio PBC, Boston, MA, USA).

## Results

### Participants

A total of 263 people took part in the study. Two participants were excluded, as they declined the declaration of consent. Another two participants were excluded because they were younger than 18 at the time of participation. Finally, 259 data sheets were included for data assessment. Detailed information about the general data of the participants concerning their diagnosis of TMD can be found in Table 1.

**Table 1:** General participants information

	n (%)	Mean (SD)	Range	Diagnosis of TMD	no Diagnosis of TMD	p-value
**Age** ^**1**^		38.8 (SD:11.0)	18–65	39.8 (SD:10.6)	37.9 (SD:11.3)	0.134
**Sex (%)**						0.642
*men*	16 (6.2)			6 (5.0%)	10 (7.2%)	
*women*	238 (91.9)			111 (92.5%)	127 (91.4%)	
*diverse*	5 (1.9)			3 (2.5%)	2 (1.4%)	
**Subtype**						0.011
*hypermobile EDS*	230 (88.8)			113 (49.1%^2^)	117 (50.9%^2^)	
*classical EDS*	29 (11.2)			7 (24.1%^2^)	22 (75.9%^2^)	
**Country (%)**						0.058
*Germany*	237 (91.5)			115 (95.8%)	122 (87.8%)	
*Austria*	7 (2.7)			1 (0.8%)	6 (4.3%)	
*Switzerland*	15 (5.8)			4 (3.3%)	11 (0.7%)	
**Time of diagnosis** ^**1**^		34.2 (SD:12.1)	1–61	36.4 (SD:11.5)	32.0 (SD:12.6)	0.223
**Time between first symptoms and diagnosis** ^**1**^		22.2 (SD:12.5)	0–55	24.6 (SD:11.8)	20.1 (SD:12.8)	0.119

General information about the participants concerning their temporomandibular disorder (TMD) diagnosis. ^1^ – in years; ^2^ – percentages are given for the number of participants concerning the subtype. P-values were calculated using a chi-square test for categorical variables or a Mann-Whitney U test for continuous variables to show whether there was an association between each variable and TMD diagnosis

### Temporomandibular disorders

#### Painful or restricted movements of the jaw

**Fig. 1:**
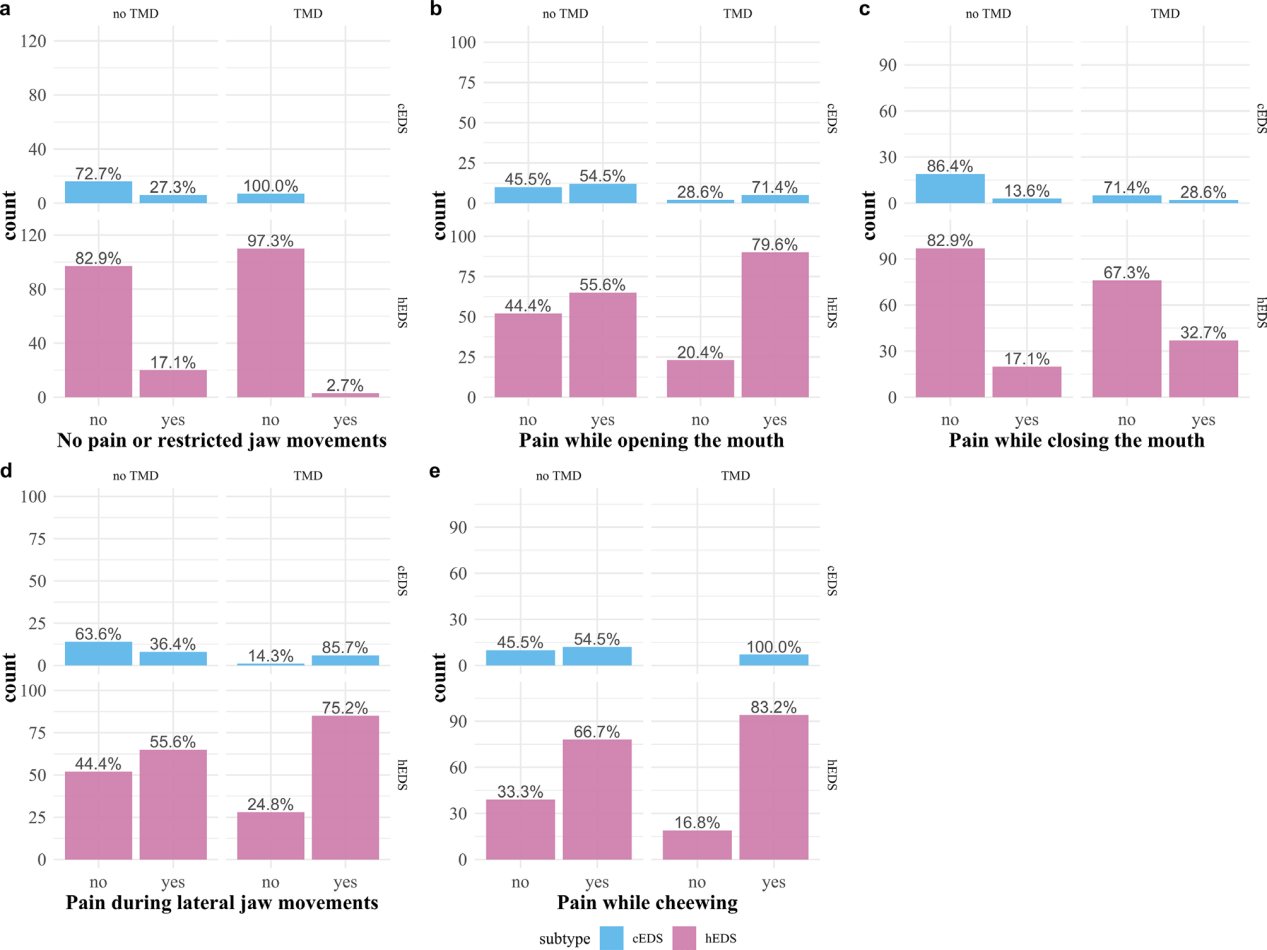
Movements of the jaw. Limited and/or painful movements of the jaw. Each figure **a–e** was separated into participants with classical EDS type (cEDS) in the upper row and hypermobile EDS type (hEDS) in the lower row. Columns were further divided for each figure into participants with and without a diagnosis of temporomandibular disorder (TMD)

**Fig. 2:**
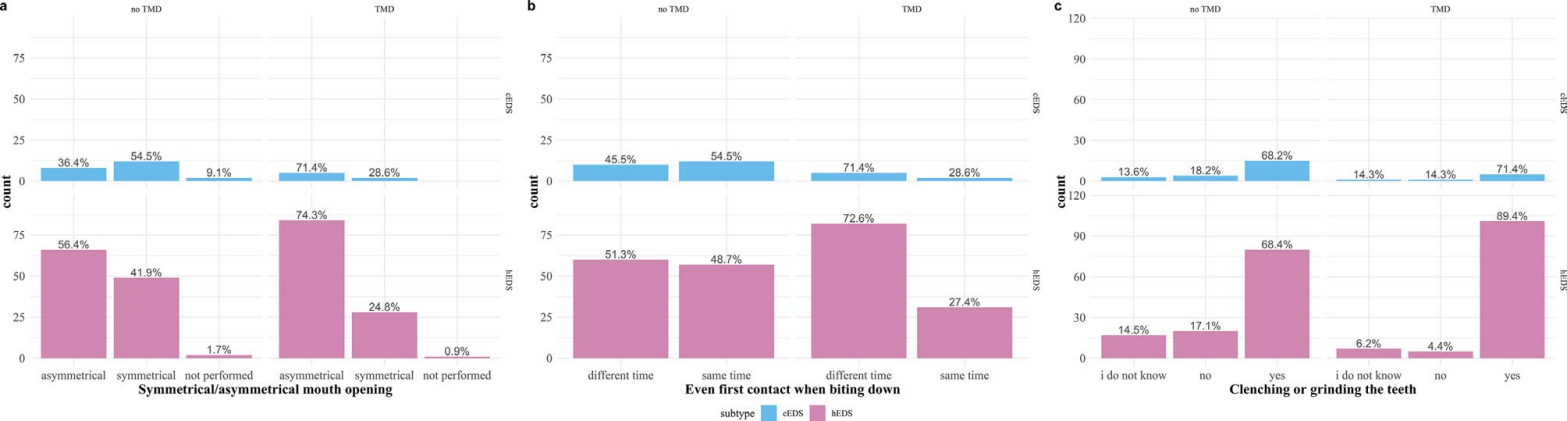
Mouth-opening movements and participants bite. Information regarding the symmetry of the mouth opening, evenness of contact, when biting down and pressing or grinding. Each individual figure **a–c** was separated into participants with classical EDS type (cEDS) in the upper row and with hypermobile EDS type (hEDS) in the lower row. Columns were further divided for each individual figure, into participants with and without a diagnosis of temporomandibular disorder (TMD)

Seventy-seven of the 259 participants with cEDS or hEDS complained of a mismatch between the size and position of the maxilla and mandible (29.7%), and 128 complained of dislocation of the temporomandibular joints (49.4%). Detailed information regarding painful or restricted jaw movements can be found in Fig. 1. For details about pressing or grinding the teeth, mouth-opening movements, and the participants’ bites, see Fig. 2.

#### Masticatory muscle pain

**Fig. 3:**
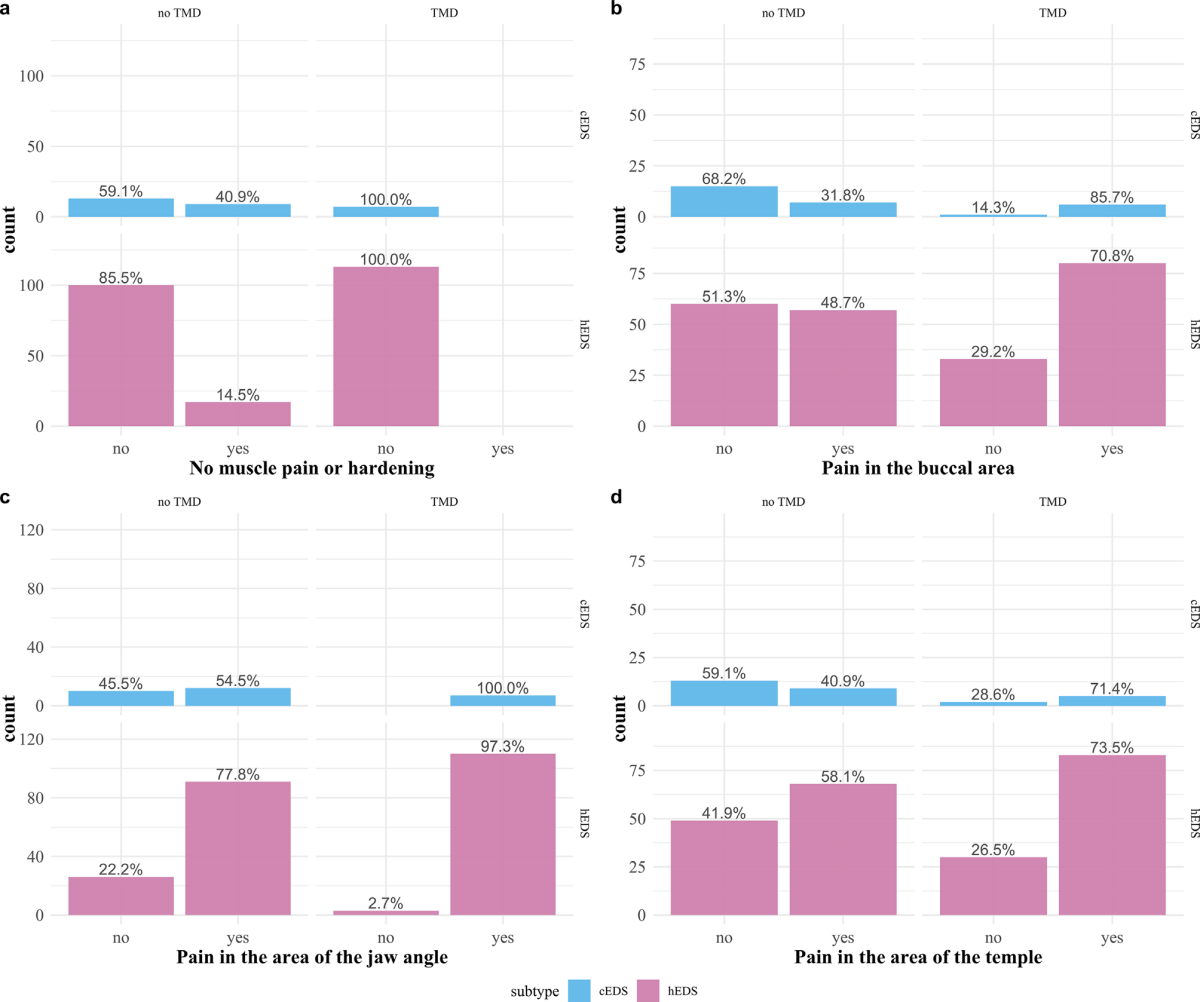
Hardening or pain of the muscles. Hardening or pain of the participants’ muscles. Each figure **a–d** was separated into participants with classical EDS type (cEDS) in the upper row and hypermobile EDS type (hEDS) in the lower row. Columns were further divided for each figure into participants with and without a diagnosis of temporomandibular disorder (TMD)

Of the 259 participants with cEDS or hEDS, 150 (57.9%) reported pain in the muscles of the cheek, 220 (84.9%) pain in the muscles of the jaw angle, and 165 (63.7%) pain of the musculature in the temporal region. Twenty-six (10.0%) participants had no pain or hardening of the muscles. Detailed information about masticatory muscle pain or hardening of the participant’s muscles can be found in Fig. 3.

#### Pain medication for TMD-specific symptoms

Fifty of the 259 (19.3%) participants with cEDS or hEDS reported using pain medication due to pain in the masticatory muscles or temporomandibular joints. Of these, 36 (72.0%) reported using it as directed by a doctor, and 14 (28.0%) reported self-medicating.

#### Diagnosis of temporomandibular disorders in both subtypes

**Fig. 4:**
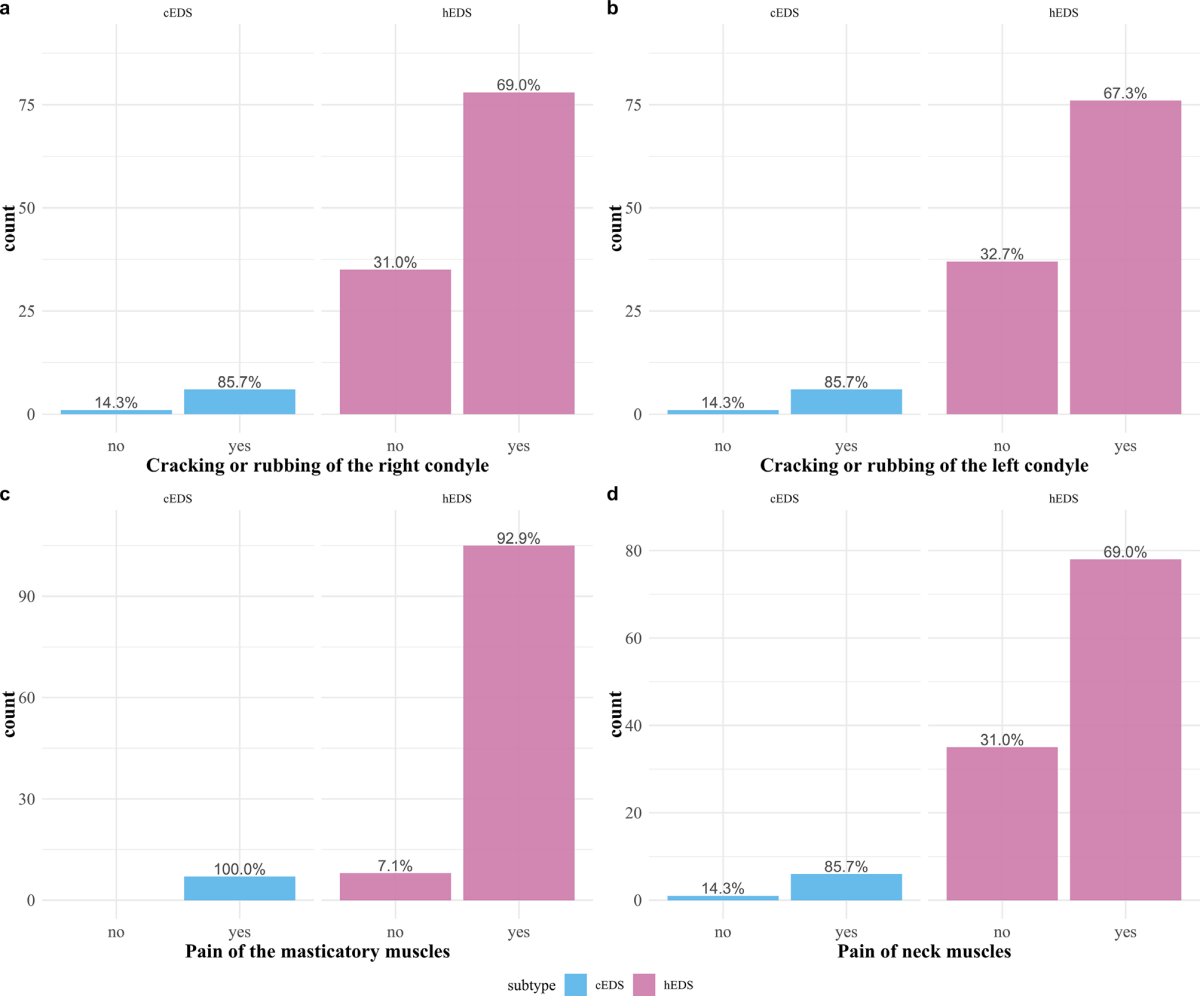
Temporomandibular disorder-specific symptoms. Information regarding the temporomandibular disorder-specific symptoms participants named. Each individual figure **a–d** was separated into participants, with classical EDS type in the left column and with hypermobile EDS type (hEDS) in the right column. Percentages were given for each subtype individually

Of the 259 participants, 120 (46.3%) reported having a diagnosed TMD (see Table 1 for TMD-diagnosis of individual subtypes). Of the 120, 84 (70.0%) reported having right TMJ clicking/grating, and 82 (68.3%) had left TMJ clicking/grating. One hundred and twelve participants (93.3%) diagnosed with TMD had pain in the masticatory muscles, and 117 (97.5%) had pain in the neck muscles. Symptoms for individual subtypes are presented in Fig. 4.

#### TMD-specific therapy

Of the 120 participants diagnosed with TMD, 86 said they had already received treatment (71.7%). Seventy-five (87.2%) by splint therapy, 49 (57.0%) by physiotherapy, two (2.3%) by acupuncture, and two (2.3%) by behavioral therapy.

**Fig. 5:**
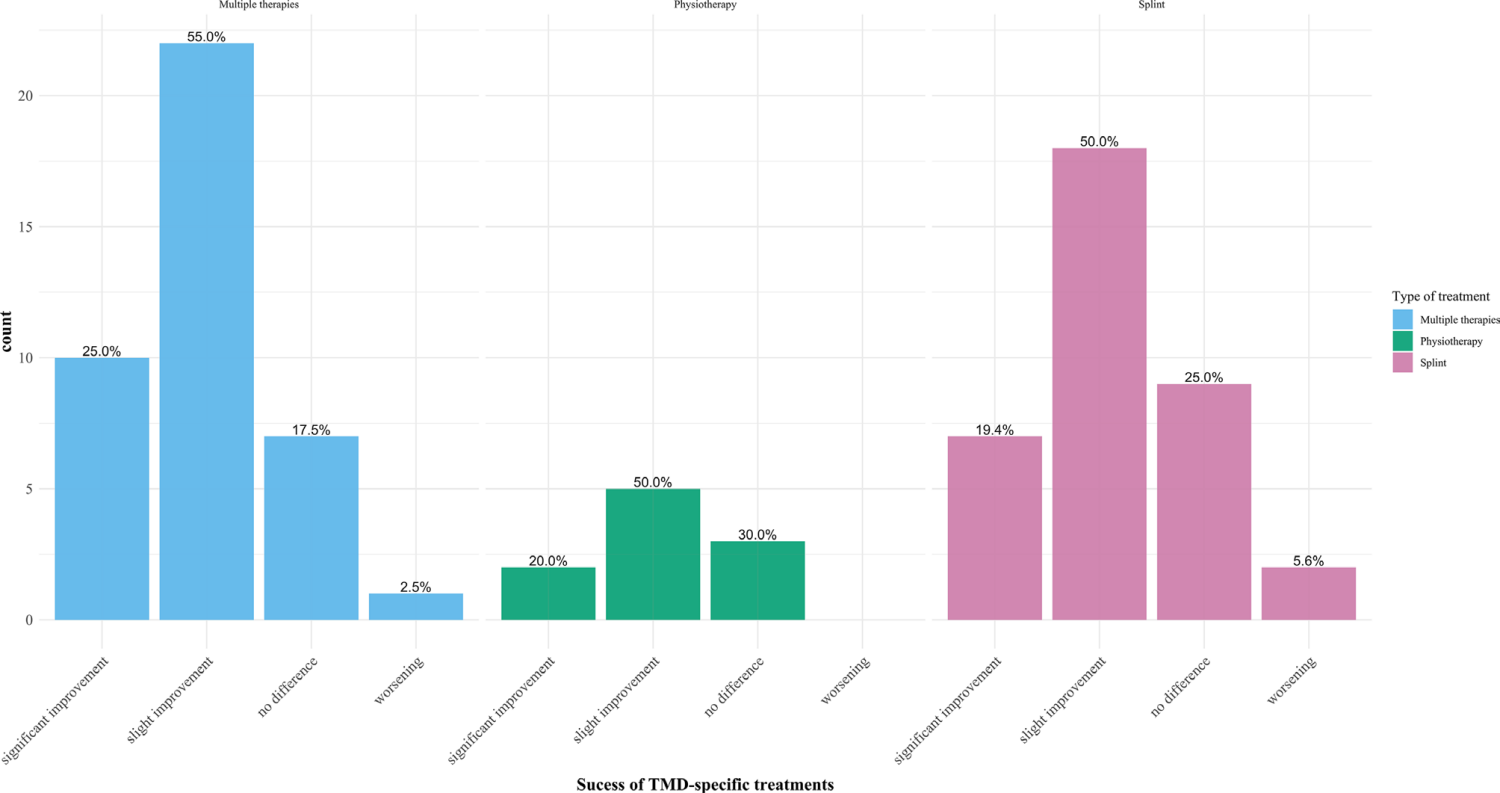
Subjectively perceived improvement through individual therapies. Information about the subjectively perceived improvement of the participants through the individual therapies. Each type of therapy was divided into four columns with the respective answer options for therapy success. Percentages were given for each type of therapy individually

Of the 86 participants who had already received treatment for diagnosed TMD, 19 reported that they had a significant improvement as a result (22.1%). Forty-five felt a slight improvement (52.3%), 19 felt no difference (22.1%), and three felt a worsening (3.5%). Information about the subjectively perceived improvement of the participants through the individual therapies can be found in Fig. 5.

### Psychological stress factors

For the 259 participants who completed the validated DASS, the following results emerged: 200 participants (20 with cEDS (69.0% of all participants with cEDS) and 180 with hEDS (78.3% of all participants with hEDS)) remained below the threshold for depression (77.2%) and 59 participants (nine with cEDS (31.0%) and 50 with hEDS (21.7%)) reached the critical value of ten (22.8%). One hundred and twenty-one participants (15 with cEDS (51.7%) and 106 with hEDS (46.1%)) remained below the critical value for anxiety (46.7%), and 138 participants (14 with cEDS (48.3%) and 124 with hEDS (53.9%)) reached the threshold of six (53.3%). In the stress category, 171 participants (22 with cEDS (75.9%) and 149 with hEDS (64.8%)) remained below the threshold (66.0%), and 88 participants (seven with cEDS (24.1%) and 81 with hEDS (35.2%)) reached the critical value of ten (34.0%). Using a chi-square test, no statistically noticeable difference in the distribution between the subtypes was found in all three categories of the DASS (P-value: depression p = 0.261, anxiety p = 0.566, stress p = 0.235).

Detailed information about the evaluation of the DASS concerning the diagnosis of the participants of TMD can be found in Table 2.

**Table 2:** Depression Anxiety and Stress Scale

n of participants with increased likelihood depression/anxiety/stress according to DASS	n	Diagnosis of TMD	no Diagnosis of DMD	p-value
*Depression*	59 (22.8%)	27 (45.8%)	32 54.2(%)	0.921
*Anxiety*	138 (53.3%)	66 (47.8%)	72 (52.2%)	0.607
*Stress*	88 (34.0%)	46 (52.3%)	42 (47.7%)	0.169

Evaluation of the Depression Anxiety and Stress Scale (DASS) concerning a diagnosed temporomandibular disorder (TMD). P-values were calculated using a chi-square test to show whether there was a relationship between the individual categories of the DASS and TMD diagnosis

### Pain-related impairment

For the 259 participants, who completed the Chronic Pain Graduation Questionnaire, it was found that 17 participants had no pain (6.6%), 135 participants had grade one (52.1%), 40 participants had grade two (15.4%), 45 participants had grade three (17.4%), and 22 participants had grade four (8.5%).

Within the subtypes, the GCS was distributed as follows: of the 29 participants with cEDS, six had no pain (20.7%), 14 had grade one (48.3%), one had grade two (3.4%), and grade three and four each had four participants (13.8% each). Of the 230 participants affected by hEDS, eleven had no pain (4.8%), 121 grade one (52.6%), 39 grade two (17.0%), 41 grade three (17.8%), and 18 had grade four (7.8%). The Chi-square test showed a significant difference in the distribution between the subtypes (p = 0.006).

Detailed information about the evaluation of the GCS concerning the diagnosis of the participants of TMD can be found in Table 3.

**Table 3:** Graduation of chronic pain

n of participants with chronic pain according to GCS	n	Diagnosis of TMD	no Diagnosis of TMD	p-value
GCS	259 (100%)	120 (46.3%)	139 (53.6%)	< 0.001
*no pain*	17 (6.6%)	0 (0.0%)	17 (100%)	
*low disability - low intensity*	135 (52.1%)	56 (41.5%)	79 (58.5%)	
*low disability - high intensity*	40 (15.4%)	23 (57.5%)	17 (42.5%)	
*high disability - moderately limiting*	45 (17.4%)	26 (57.8%)	19 (42.2%)	
*high disability - severely limiting*	22 (8.5%)	15 (68.2%)	7 (31.8%)	

Evaluation of the questionnaire for the graduation of chronic pain, “Graduierung chronischer Schmerzen” (GCS), concerning a diagnosed

temporomandibular disorder (TMD). P-values were calculated using a chi-square test to show whether there was an association between the evaluation of the GCS and TMD diagnosis

### Multivariate analysis

**Fig. 6:**
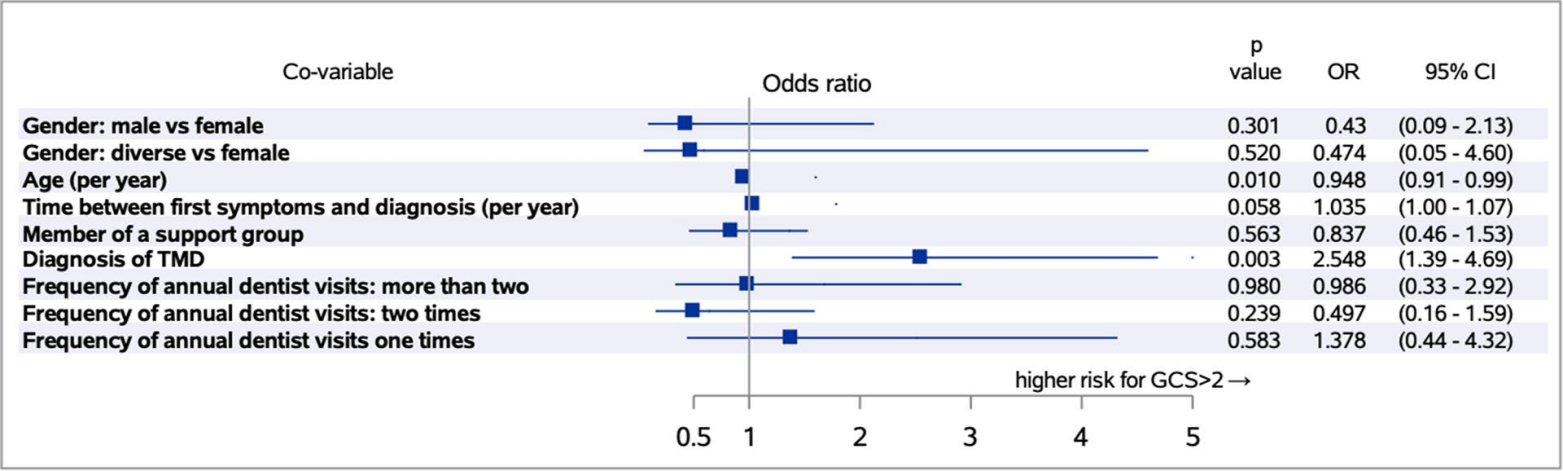
Multivariable logistic regression analysis. Multivariable logistic regression analysis of the questionnaire for the graduation of chronic pain, “Graduierung chronischer Schmerzen” (GCS), concerning gender, age, the time between first symptoms and diagnosis, member of a support group, diagnosis of temporomandibular disorder (TMD) and frequency of annual dentist visits

A threshold value for the GCS > 2 (dysfunctional pain) was chosen. The analysis shows that the risk of being categorized as a chronic pain patient decreases slightly with each year of life (p = 0.010). People who are already diagnosed with TMD show a 2.5-fold increased risk of chronic pain, i.e., having pain in the category of “high disability - moderately limiting” or “high disability - severely limiting” concerning the GCS (p = 0.003). The multivariate analysis can be seen in Fig. 6.

## Discussion

This study aimed to evaluate patients with EDS regarding temporomandibular disorder, chronic pain, and psychological distress. Because EDS represents a heterogeneous clinical picture, the prevalence varies significantly between subtypes. As the previous studies [12, 23, 26], most patients in our cohort were diagnosed with the hypermobile subtype (88.8%).

One of the typical symptoms of persons affected by EDS is hypermobility of the joints [8, 12, 16, 27]. In the oral region, this often involves the temporomandibular joint. This hypermobility can lead to temporomandibular disorder or classic TMD symptoms [28–30]. Thus, in our cohort, nearly half of the participants (46.3%) had TMD already diagnosed. In addition, an even higher proportion reported having specific TMD symptoms. Only 11.2% of the participants reported having no pain at all with temporomandibular joint movements, and even only 10.0% of the participants reported having no pain at all in the muscles in the oral region. In comparison, in a meta-analysis to determine the prevalence of treatment need for TMD in the average adult population (from 19 to 78 years), Al-Jundi et al. 2008 found that 15.6-16.2% of adults had a treatment need for TMD [31]. This shows the high prevalence of TMD in our cohort or the increased demand for treatment for TMD in patients with EDS.

Compared to our cohort, previous studies about EDS found similar results, although they never asked about already diagnosed TMD. For example, Hagberg et al. showed in a questionnaire study of temporomandibular joint problems and mandibular opening skills in patients with EDS that almost half of the participants had permanent problems with the temporomandibular joints (44.0%) or masticatory muscles (50.0%) (ref [12]). Also, nearly half (50.0%) of the participants described frequent grinding or clenching of the teeth. De Coster et al. [32] demonstrated in a study of oral health in patients with EDS that all participants with EDS had additional TMD symptoms. Recent results of study by Fairweather et al. [33] have shown that jaw pain is significantly more common in individuals with hEDS or HSD when the patients are also diagnosed with fibromyalgia (87.7% of patients when both diagnoses are present) compared to a control group that is neither hypermobile nor affected by fibromyalgia. Interestingly, no significant difference was found between the hEDS/HSD group (without fibromyalgia) and the control group in this study, suggesting that further studies are needed to investigate the relationship of fibromyalgia, hEDS/HSD and TMD to understand the significance and impact of fibromyalgia on TMD.

TMD is probably underdiagnosed in EDS since, in our cohort, noticeably more participants answered the TMD-specific symptoms in the affirmative than the question of whether an already diagnosed TMD was presented.

Due to the not uncommon lack of knowledge about the frequent occurrence of TMD in patients with EDS and the associated non-treatment of these, general medical problems, as well as the chronicity of pain, could also occur in the long term to the typical symptoms [6, 11, 34]. These findings inevitably raise the question of adequately treating patients suffering from EDS concerning their symptomatology. Mitakides and Tinkle [35] stated that for prevention of TMD problems, the prophylactic therapy recommendation in all EDS patients was the treatment of postural and upper and lower back problems and lifestyle modification of masticatory patterns, diet, stress reduction, and physical activity. Standard non-invasive therapies for TMD patients without EDS included splint and massage therapy (physiotherapy), light and laser therapy, and drug therapy [36–38].

Since little is known about the therapeutic success of these methods in patients with EDS, future research should investigate more closely if the respective therapies affect patients with EDS to come closer to a suitable treatment with long-term success and to develop a guideline. In our cohort, 33.0% of the total participants, or 71.7% of the participants with a previously diagnosed TMD, reported already receiving TMD-specific therapy. The vast majority, through splint therapy, a smaller proportion utilize physical therapy. In addition, most participants reported feeling at least moderate improvement due to the treatment. Hagberg et al. [12] demonstrated that patients with EDS and TMD symptoms were significantly more likely to use analgesic or hypnotic medications than non-EDS individuals and showed that only a small proportion was treated by splint therapy. This heterogeneity in the results about treating persons suffering from EDS with TMD or TMD-specific symptoms demonstrates the lack of a clear line and further research concerning this topic. It illustrates the problem of treating these patients very well. Thus, the difficulty in care arises simply from the fact that TMD is not caused by acquired dysfunction or similar causes but rather arises conditionally from an underlying disease that cannot be cured and the resulting hypermobility. Hence, no reason can be remedied. Therefore, exploring therapies that bring success, even without dysfunction, is even more critical. Thus, patients with EDS should be clinically evaluated for TMD to verify these findings.

One consequence of TMD in patients with EDS is the development of pain, especially chronic pain in the long term [34]. After evaluation of the collected data, a minor proportion of participants in our cohort (6.6%) had no pain; of these 6.6%, none had a diagnosed TMD. An evidence-based comparison to the average population is difficult. However, an observed proportion of ¼ of participants with dysfunctional chronic pain is remarkable. As the level of chronic pain increases, the percentage of participants, who report already being affected by TMD, also rises, and a significant association between the diagnosis of TMD and the severity or chronicity of the pain was shown. The multivariable analysis revealed a 2.5-fold increased risk for dysfunctional chronic pain for patients diagnosed with TMD. Future research should consider if early and perhaps even prophylactic treatment of TMD in patients with EDS can prevent the development or chronification of pain, at least in the masticatory area. Syx et al. conducted a study on EDS and chronic pain and found that patients with EDS are often affected by chronic pain, especially patients with hEDS [8]. In addition, it was found that chronic pain in patients with EDS is often poorly treated with conventional analgesics and physiotherapy. The reasons suggested were nociceptive pain directly due to structural changes in the affected joints, muscles, and connective tissues, neuropathic pain, impaired proprioception, muscle weakness, and central sensitization [8]. Further research should investigate the possible role of co-occurrence of TMD and chronic pain as a clinical marker for hEDS and cEDS.

The manifestation of chronic pain goes hand in hand with a long time between the onset of symptoms and diagnosis in individuals affected by rare diseases. Schmitt-Sausen [39] stated that the period between the onset of symptoms and diagnosis in rare diseases is about seven years. The average in our cohort was 22.2 years (SD: 12.11). The resulting pain can also become chronic and affect general health. The multivariable analysis of this study showed that, among other things, delay in diagnosis increased the likelihood of severe pain. As stated by Kalisch et al., the delay in diagnosis may be partly responsible for the high pain burden of people suffering from EDS [40]. In future research, it is essential to prioritize early diagnosis in patients with EDS or all patients with rare diseases. The importance of this was shown in 2019 by Bohner et al., who found a decreased OHRQoL with each year a patient is waiting for a diagnosis [41].

Symptoms without knowing the origin or a confirmed diagnosis can also cause psychological distress. Niemeyer et al. [42] showed that people with EDS had clinically significant anxiety and depressive symptoms. They also found that pain intensity correlated significantly with depression but not anxiety intensity. Also, in our cohort, about 20-30% of the participants had depression symptoms or severe stress, and almost half had anxiety symptoms. Hershenfeld et al. [26] showed an association between chronic pain and psychiatric diagnoses in patients with EDS but also no association between joint hypermobility and psychiatric diagnoses in people with EDS. Joint hypermobility is a prevalent symptom of TMD. Future studies should continue investigating the relationship between chronic pain and psychological distress in patients with EDS.

### Limitations

The patient population is a common problem in studies dealing with rare diseases. Like a rare disease, only a few people are affected. This creates several difficulties in collecting sufficient data. A commonly described problem in EDS studies is the unbalanced gender distribution. In our cohort, only 6.2% of the participants were male patients. This distribution is consistent with previous studies, which describe a significant majority of female participants [12, 22, 23, 26, 34, 43]. A possible reason for this imbalance could be that women organize themselves more often in self-help groups and participate more often in studies about their disease.

Another problem of the study conducted is online distribution and restriction to Germany, Austria, and Switzerland. This meant that only subjects with a working internet connection could participate. If one assumes that the younger generation nowadays mostly has internet access, it must be assumed that older people without internet may not be included.

A further problem in studies that collect data with an anonymous questionnaire filled out by the participants is that the patient’s subjective feelings are very strongly reflected and some diagnosis such as hEDS and cEDS as well as TMD can only be collected through self-report. Even if a medically confirmed diagnosis is explicitly asked for, the participants must be trusted in this context. In future research, these results should be clinically tested and verified. Our and similar studies should be used as a basis for this.

## Conclusions

There was a high prevalence of TMD problems and chronic pain in patients with cEDS and hEDS. Furthermore, there was a significant association between these two diagnoses, and the presence of TMD increased the risk of chronic pain. Currently, there is no adequate treatment for these issues in patients with cEDS and hEDS, nor guidelines for managing TMD and chronic pain in EDS. In addition, late diagnosis is an issue that, combined with the lack of knowledge about adequate treatment, exacerbates these issues and creates high psychological distress.

Overall, the results reflect that further studies urgently need to investigate how far chronic pain and TMD in patients with EDS can be adequately treated conventionally or with medication. This study showed that most of the cohort experienced an improvement in symptoms and chronic pain through TMD therapy. However, this needs to be verified clinically through further studies. Thus, there is a need for further research into drug treatment and conventional therapy for EDS patients. To relieve patients of the high level of suffering and psychological distress caused by their disease, future research should focus on how people with EDS can be diagnosed early and thus receive early and adequate, possibly prophylactic, therapy. This could minimize the consequences, such as chronic pain and psychological diagnoses.

### Electronic supplementary material

Below is the link to the electronic supplementary material.

Supplementary Material 1

## Data Availability

All data from this study are presented within the results section or are available from the corresponding authors upon reasonable request.
