# Supplementary material for: Prevalence and quality of temporomandibular disorders, chronic pain and psychological distress in patients with classical and hypermobile Ehlers-Danlos syndrome: an exploratory study
Source: Orphanet J Rare Dis. 2023 Sep 19;18:294. doi: 10.1186/s13023-023-02877-1 (PMC10510186; doi:10.1186/s13023-023-02877-1)
Supplement: Supplementary file 1 — Supplementary Material 1 [file 13023_2023_2877_MOESM1_ESM.docx]

# Supplementary File S1 – Translated online questionnaire

1. **Gender**

- Male
- Female
- Diverse

1. **Age**

*The age was entered as free text.*

1. **In which country do you live?**

- Germany
- Austria
- Switzerland

1. **Subtype**
   **Either a clinical or human genetic diagnosis must have been made. The subtypes are based on the International Classification of Ehlers-Danlos Syndromes from 2017**

- Classical EDS (cEDS)
- Hypermobile EDS (hEDS)

1. **How old were you when you were first diagnosed with your disease?**

*The age was entered as free text.*

1. **How much time has elapsed between the first appearance of symptoms and the definitive diagnosis of your disease?**

*The number of years were given as free text.*

1. **Are you a member of a support group and/or do you attend support group meetings?**

- Yes
- No

1. **How often have you seen a dentist within the last 12 months?**

- Not once
- Once
- Twice
- More often than twice

1. **Are there currently, or have there been in the past, one or more of the following movements that are limited and/or painful?**
   (Multiple answers are possible)

- No
- Chewing
- Mouth opening
- Mouth closure
- Lateral jaw movements

1. **When you open your mouth slowly, does it open straight (symmetrical) or does it deviate to one side (asymmetrical)?**
   **Please use a mirror for this purpose or have someone else examine the mouth opening. Pay particular attention to the movement of the incisors and repeat the opening and closing movement several times.**

- Not feasible
- Symmetrical
- Asymmetrical

1. **Do you currently have, or have you had in the past, hardening and/or pain of the muscles in one or more of the following locations?**
   (Multiple answers are possible)

- No pain or hardening
- Buccal area
- Area of the jaw angle
- Area of the temple

1. **When you bite down slowly and in a controlled manner, do you feel that your upper and lower molars meet at the same time or do individual teeth touch first before full contact can be made?**

- Rather simultaneous
- Rather first individual teeth

1. **Do you clench your teeth and/or grind them during the day and/or during the night? Have you been made aware of this by sleep partners, if applicable?**

- Yes
- No
- I don’t know

1. **Have you been diagnosed with temporomandibular dysfunction (TMD)?**
   **Temporomandibular dysfunction, or TMD for short, is the term used to describe a painful dysregulation of the masticatory system. It can be triggered by a disturbed interaction of various muscles and joints.**

- Yes
- No

*The following questions “15” And “16” could only be answered if the previous question “14” was answered with “Yes”.*

1. **What TMD-specific symptoms have you been diagnosed with?**
   **(Multiple answers are possible)**

- Cracking/grating of the right temporomandibular joint
- Cracking/grating of the left temporomandibular joint
- Pain of the masticatory muscles
- Pain of the neck muscles
- Other symptoms

1. **Are you currently receiving treatment for TMD?**
   **e.g. in the form of splint therapy, physiotherapy etc.**

- Yes
- No

*The following questions “17”, “18” and “19” could only be answered if the previous question “16” was answered with Yes.*

1. **In what form is CMD currently being treated?**
   **(Multiple answers are possible)**

- Splint therapy
- Physiotherapy
- Acupuncture
- Behavioral therapy
- Ergotherapy
- Other type of treatment

1. **Do you feel any improvement as a result of the treatment?**

- Yes, a significant improvement
- Yes, a slight improvement
- I do not feel any difference
- No, I feel a worsening

1. **Are you currently taking pain medication for pain of the masticatory muscles or temporomandibular joints?**

- Yes
- No

*The following question “20” could only be answered if the previous question “19” was answered with Yes.*

1. **Are you taking the medication as directed by a doctor or as self-medication?**

- On the order of a doctor
- Self-medication

*The following questionnaire “21” represents the German version of the Depression Anxiety and Stress Scale. For each question, the corresponding answer was selected from one of the 4 possibilities.*

1. **Questions about how you felt during the last week.**
   **Please read each statement and tick the one that applies to you. There is no right or wrong answer, try to decide spontaneously for one answer.**

|  | **Did not apply to me at all**  **-**  **Never** | **Applied to me to a certain extent**  **-**  **Sometimes** | **Applied to me to a considerable extent**  **-**  **Quite often** | **Applied very strongly to me**  **-**  **Most of the time** |
| --- | --- | --- | --- | --- |
| I found it hard to calm down. |  |  |  |  |
| I felt that my mouth was dry. |  |  |  |  |
| I could no longer experience any positive feelings at all. |  |  |  |  |
| I had breathing problems (e.g. excessively rapid breathing). |  |  |  |  |
| I had a hard time getting myself to get things done. |  |  |  |  |
| I tended to overreact to situations. |  |  |  |  |
| I was shaking (e.g., hands) |  |  |  |  |
| I found everything exhausting. |  |  |  |  |
| I worried about situations where I might panic and make a fool of myself. |  |  |  |  |
| I felt like I had nothing to look forward to. |  |  |  |  |
| I noticed that I was getting upset quickly. |  |  |  |  |
| I found it difficult to relax. |  |  |  |  |
| I felt depressed and sad. |  |  |  |  |
| I reacted indignantly to everything that prevented me from continuing my current activity. |  |  |  |  |
| I felt close to panic. |  |  |  |  |
| I was not able to get excited about anything. |  |  |  |  |
| I didn't feel like I had much value as a person. |  |  |  |  |
| I found myself quite sensitive. |  |  |  |  |
| I felt my heartbeat without physically exerting myself (e.g., feeling my heart racing). |  |  |  |  |
| I felt anxious for no reason. |  |  |  |  |
| I felt that life was meaningless. |  |  |  |  |

*The following questionnaire (“22” to “24”) represents the German version of the Graded Chronic Pain Status (GCPS), "Graduierung chronischer Schmerzen" (GCS).*

*The first question was answered as free text. For the following 6 questions, one of the 11 options from 0 to 10 was selected.*

1. **Approximately how many days in the past 6 months have you been unable to perform your normal activities (job,school,study,chores) because of your facial pain?**

*The days were entered as free text.*

1. **The next three questions, are about the severity of your facial pain. Please mark your score on a scale from 0 to 10.**
   **0 = no pain
   10 = pain could not be worse**

|  | **0** | **1** | **2** | **3** | **4** | **5** | **6** | **7** | **8** | **9** | **10** |
| --- | --- | --- | --- | --- | --- | --- | --- | --- | --- | --- | --- |
| How would you classify your pain in the facial area as you are at this moment? |  |  |  |  |  |  |  |  |  |  |  |
| How would you rank your most severe facial pain in the past 6 months? |  |  |  |  |  |  |  |  |  |  |  |
| How would you rate the average severity of facial pain over the past 6 months? |  |  |  |  |  |  |  |  |  |  |  |

1. **The next three questions, are about how much facial pain interferes with activities.**

**Please mark your score on a scale of 0-10.**
**0 = no impairment**

**10 = You are/were unable to do anything**

|  | **0** | **1** | **2** | **3** | **4** | **5** | **6** | **7** | **8** | **9** | **10** |
| --- | --- | --- | --- | --- | --- | --- | --- | --- | --- | --- | --- |
| In the past 6 months, to what extent has your facial pain affected you in your daily activities? |  |  |  |  |  |  |  |  |  |  |  |
| In the past 6 months, to what extent has facial pain affected your ability to participate in family or free-time activities? |  |  |  |  |  |  |  |  |  |  |  |
| In the past 6 months, to what extent has facial pain affected your ability to do your work/homework? |  |  |  |  |  |  |  |  |  |  |  |
